# Beyond Psychological Trauma: Associations of Nutritional Status with Depression in Child and Adolescent Victims of Crime

**DOI:** 10.3390/healthcare14081075

**Published:** 2026-04-17

**Authors:** Ahmet Depreli, Emre Adıgüzel, Burcu Çavdar, Fatma Coşkun

**Affiliations:** 1 Konya Forensic Medicine Branch Directorate, Council of Forensic Medicine, 42060 Konya, Türkiye; ahmetdep@gmail.com; 2 Department of Nutrition and Dietetics, Faculty of Health Sciences, Karamanoğlu Mehmetbey University, 70100 Karaman, Türkiye; 3 Non-Communicable Diseases Unit, Department of Public Health, Tokat Provincial Health Directorate, 60030 Tokat, Türkiye; burcucavdr@gmail.com; 4 Department of Child and Adolescent Psychiatry, Meram Medical School, Necmettin Erbakan University, 42090 Konya, Türkiye; mdfcoskun@gmail.com

**Keywords:** childhood trauma, criminal victimization, depression, eating behavior

## Abstract

**Highlights:**

**What are the main findings?**

- Depression severity in child and adolescent victims of crime was strongly associated with adverse nutritional status, including higher body weight, BMI Z-score, body fat percentage, and greater dietary energy and macronutrient intake.
- Poor diet quality—particularly low adherence to the Mediterranean diet and insufficient vitamin C and fiber intake—was associated with depressive symptom severity.

**What are the implications of the main findings?**

- Nutritional status may be an important factor to consider in the assessment of depression in child and adolescent victims of crime, alongside psychological and psychiatric evaluation.
- Integrating nutrition-focused interventions, such as improving diet quality and promoting healthy eating behaviors, may enhance mental health outcomes and support long-term well-being in traumatized children and adolescents.

**Abstract:**

Background/Objectives: Children and adolescents exposed to criminal victimization are at increased risk for depression; however, the contribution of nutritional status to depressive symptom severity in this vulnerable population remains poorly understood. Therefore, we aimed to examine the associations between depression severity and nutritional parameters in child and adolescent victims of crime. Methods: This cross-sectional study included 72 children and adolescents (aged 10–16 years) referred to a forensic medicine department in Türkiye. Nutritional status was assessed using anthropometric measurements (body weight, body mass index [BMI], BMI-Z score, and body fat percentage), three-day dietary records, and the Mediterranean Diet Quality Index (KIDMED). Depression severity was evaluated using the Kutcher Adolescent Depression Scale (KADS). The associations were analyzed using Pearson’s rho correlation and forward stepwise linear regression. Potential confounding variables, including age, gender, socioeconomic status, and trauma-related characteristics, were recorded and considered during the analysis; however, due to the limited sample size and to avoid model overparameterization, they were not fully adjusted for in the final model. Results: Depression severity was positively correlated with the body weight, BMI, BMI-Z score, body fat percentage, and dietary energy, carbohydrate, protein, and fat intakes (all *p* < 0.05). In contrast, the vitamin C and dietary fiber intakes, breastfeeding duration, and KIDMED scores were negatively correlated with the KADS scores (*p* < 0.05). Regression analysis revealed that the lower KIDMED scores, higher body fat percentage, and greater body weight were significantly associated with depression severity, collectively explaining 82.2% of the variance in the KADS scores. Conclusions: Poor diet quality and adverse body composition are strongly associated with depression severity in child and adolescent victims of crime. These findings suggest that nutritional factors may be associated with depression severity in child and adolescent victims of crime; however, the results should be interpreted as preliminary and hypothesis-generating.

## 1. Introduction

Individuals who are exposed to a crime and suffer physical, psychological or social harm are called “victims” [1]. Child victims of crime are defined as children and adolescents who are directly exposed to maltreatment, such as intentional or unintentional physical or sexual abuse or neglect. The weak and fragile nature of children makes them vulnerable to neglect and abuse [2]. Childhood and adolescence are the most sensitive stages of life, during which the basis of cognitive and physical development is laid and the most care and attention are required. Therefore, negative experiences in childhood can have lasting effects on the developmental process [3]. The basis of many mental problems encountered in adulthood is laid in childhood and adolescence, where traumatic events experienced during childhood can seriously affect children’s mental health and quality of life, leading to many social, psychological, behavioral, and biological problems in later life. In children exposed to traumatic experiences, many problems such as withdrawal, social communication disorders, disturbances in sleep quality, anxiety, anger outbursts, decreased academic performance, lack of self-confidence, substance abuse, and eating disorders can be observed [4,5,6].

Trying to cope with feelings of guilt and shame in victimized children can lead to stress disorders. Anxiety and irritability are also acute reactions that often accompany stress. More importantly, for children, being a victim of crime can elicit feelings of insecurity, worthlessness, and guilt, leading to depression [7,8,9]. Childhood depression is a common, recurrent, and chronic condition characterized by mental breakdown and loss of interest and enthusiasm. Studies have shown a strong relationship between victimization from crimes and problems such as childhood depression, anxiety, eating disorders, substance abuse, and suicide. Child victims of crime may have negative thoughts about the future. Children who experienced abuse and neglect may have various behavioral and emotional disorders such as low self-esteem, hopelessness, and fear of abandonment [10,11]. Assessments of the sociodemographic characteristics and mental conditions of child victims of crime are critical for understanding the extent and effects of the traumas [12].

While a substantial body of literature has examined the psychological consequences of childhood trauma, these studies have largely focused on psychiatric and behavioral outcomes [13]. In parallel, a growing body of evidence indicates that nutritional factors, such as diet quality, micronutrient intake, and obesity, are associated with depressive symptoms in children and adolescents. For instance, adherence to healthy dietary patterns, such as the Mediterranean diet, has been linked to lower depressive symptoms [14], whereas diets high in processed foods, saturated fats, and added sugars have been associated with an increased depression risk in youth [15]. In addition, obesity has been consistently associated with depressive symptoms in children and adolescents, although the directionality of this relationship remains complex [16]. Furthermore, several micronutrients involved in neurotransmitter synthesis and neurobiological regulation, such as vitamins and dietary fiber, have been implicated in mood regulation [17,18]. Therefore, healthy eating is known to play an important role in growth, development, and physical health, as well as in cognitive function and mental health. Inadequate and unbalanced nutrition may adversely affect the endocrine, immune, and nervous systems, particularly in developing children and adolescents [19]. Moreover, nutrients essential for brain function may influence mood, behavior, and cognitive processes through their role in neurotransmitter synthesis [20]. However, current evidence on the relationship between nutrition and mental health has predominantly been derived from general or non-clinical populations. Importantly, studies specifically examining the association between nutritional status and depression in child and adolescent victims of crime are extremely limited. This represents a critical gap, as this population is characterized by both heightened psychological vulnerability and potentially disrupted eating behaviors. Therefore, the present study aims to examine the associations between nutritional status and depression severity in child and adolescent victims of crime. As such, this study sought to contribute to the literature by providing a more integrative perspective that considers both psychological and nutritional dimensions of depression in a highly vulnerable population.

## 2. Materials and Methods

### 2.1. Subjects and Ethics

The study was conducted on children and adolescents aged 10–16 who were victims of crime and were referred to the Tokat Forensic Medicine Branch Directorate by the Tokat Chief Public Prosecutor’s Office. Between May and November 2024, 116 children/adolescents and their families were interviewed at the Tokat Forensic Medicine Branch Directorate, and 72 children and adolescents whose parents agreed to participate in the study were enrolled. The participants were recruited consecutively from all children and adolescents referred to the Tokat Forensic Medicine Branch Directorate during the study period. No additional sampling or selection procedures were applied beyond eligibility criteria and consent. The parents of the participants signed the voluntary participation form. The Declaration of Helsinki (World Medical Association) was considered in all data collection procedures.

The study was approved by the Tokat Gaziosmanpaşa University Clinical Research Ethics Committee (approval number: 83116987-248, date: 21 December 2023). In addition, a study permit was obtained from the Ministry of Justice (Türkiye) Council of Forensic Medicine (approval number: 21589509/2024/142, date: 30 April 2024).

### 2.2. Procedure

Descriptive characteristics of the participants (age, gender, household income, type of criminal act suffered, loss of a parent, parental cohabitation status, family history of crime, and duration of breast milk intake) were collected with a general questionnaire. To assess nutritional status, anthropometric measurements (height, body weight, body mass index (BMI), and BMI-Z score) were taken by well-trained researchers and a three-day dietary record was obtained. In addition, the Mediterranean Diet Quality Index (KIDMED) was used to assess healthy eating behavior. Finally, the Kutcher Adolescent Depression Scale (KADS) was used to assess depressive symptoms. All data were collected by face-to-face interview. The inclusion criteria were as follows: (i) being aged between 10 and 16 years, (ii) being referred as a victim of crime, and (iii) providing informed consent from parents or legal guardians. The exclusion criteria included the following: (i) presence of chronic diseases affecting nutritional status, (ii) use of medications influencing appetite or metabolism, and (iii) incomplete dietary or questionnaire data.

### 2.3. Measures

#### 2.3.1. Anthropometric Measurements

Height and body weight measurements were performed in accordance with standard methods. Height was measured without shoes and accessories such as hairpins, crowns, and hats, and with the head in the Frankfort plane in an upright position. A wall-mounted stadiometer sensitive to 0.1 cm was used for the measurements. Body weight and fat percentage were measured using a calibrated bioelectrical impedance analysis (BIA) device (Tanita BC 730, Tanita Corp., Tokyo, Japan) sensitive to 0.1 kg, with participants wearing light clothing and no shoes. The measurements were performed under standardized conditions, with participants in a fasting state and having avoided intense physical activity prior to assessment. The equation “BMI = body weight (kg)/height (m^2^)” was used to calculate the body mass index [21]. Since the sample of the study consisted of children and adolescents, BMI-Z scores were calculated. The WHO AnthroPlus program, which was prepared according to the World Health Organization (WHO) 5–19 age growth references, was used to determine the BMI-Z scores.

#### 2.3.2. Dietary Intake

Three consecutive days of dietary records were taken to determine the average daily energy and nutrient intakes. A photographic food and meal atlas, the “Food and Meal Photo Catalog: Measurements and Quantities)”, was used to determine the portion sizes [22]. The obtained data were analyzed using the Nutrition Information System (version 9.0) [23], which is food analysis software. Thus, the average daily intakes of energy (kcal), carbohydrate (g), protein (g), fat (g), β-carotene (µg), retinol (µg), vitamin D (µg), vitamin E (µg), folate, vitamin B_12_, vitamin C (mg), calcium (mg), iron (mg), and dietary fiber (g) were determined for each participant.

To improve the accuracy of the dietary records, participants and their caregivers were provided with detailed instructions on how to record food intake. Dietary records were reviewed together with the participants during face-to-face interviews by trained researchers to verify the completeness and clarify portion sizes. When necessary, probing questions were used to minimize underreporting or overreporting.

#### 2.3.3. Mediterranean Diet Quality Index (KIDMED)

The Mediterranean Diet Quality Index (KIDMED), which aims to assess healthy eating habits and adherence to the Mediterranean diet, was developed by Serra-Majem et al. [24]. The scale consists of 16 items, 12 items are positive statements, 4 items are negative statements; all items are answered as “yes” or “no”. For all items, answering “no” corresponds to 0 points; answering “yes” to each positive statement corresponds to +1 point, while answering “yes” to each negative statement corresponds to −1 point. Summing the item scores results in scores ranging from −4 to 12. A score of ≤3 points indicates a low level of adherence to the Mediterranean diet, a score of 4–7 points indicates a moderate level of adherence, and a score of ≥8 points indicates a high level of adherence. The Turkish validity and reliability study of the scale was conducted by Akar-Şahingöz et al. [25]. Within the scope of this study, Cronbach’s alpha internal consistency coefficient for the KIDMED was found to be 0.917.

#### 2.3.4. Kutcher Adolescent Depression Scale (KADS)

The KADS, which comprises 11 items measured on a 4-point Likert scale, was developed by Brooks and Kutcher [26] to assess the severity of depression in adolescents. Each item corresponding to depressive symptoms is scored across 0–3 points (almost never = 0, sometimes = 1, often = 2, always = 3). Higher scores on the scale indicate a more severe depressive mood. The Turkish validity and reliability study of the scale was conducted by Balcı-Çelik and Uysal-Atabay [27]. Within the scope of this study, the Cronbach’s alpha internal consistency coefficient for the KADS was found to be 0.966.

### 2.4. Statistical Analyses

Statistical analyses were performed using the Statistical Package for Social Sciences (SPSS) version 25.0. The Shapiro–Wilk test, skewness/kurtosis values, and histogram graphs were considered to evaluate normality. Variables that meet the normality conditions according to at least two of these three criteria were considered to be normally distributed. The range of −1 to +1 for skewness/kurtosis values was considered as an indicator of normality. The relationships between KADS scores and nutritional parameters (anthropometric measurements, dietary energy and nutrient intakes, duration of breast milk intake, and KIDMED scores) were evaluated using Pearson’s rho correlation. In addition, linear regression was used to examine factors associated with KADS scores among the following candidate variables: body weight, BMI-Z score, body fat percentage, dietary energy, carbohydrate, protein, fat, duration of breastmilk intake, and KIDMED scores. A forward stepwise approach was preferred to reduce the risk of overparameterization given the relatively small sample size and the number of candidate predictors, and to obtain a more parsimonious model by retaining only the most relevant variables. At each step, variables were added based on their *p*-values, and a *p*-value threshold of 0.100 was used to set a limit on the total number of variables included in the final model. Potential confounding variables (e.g., age, gender, household income, and trauma-related characteristics) were recorded and considered during the analysis; however, only variables meeting the predefined statistical criteria were included in the final regression model.

To assess the robustness and stability of the regression model and address potential overfitting due to the relatively small sample size, additional sensitivity analyses were conducted. First, multicollinearity diagnostics were evaluated using variance inflation factor (VIF) and tolerance values. Second, adjusted R^2^ values were reported to account for the model complexity. Furthermore, a bootstrap resampling procedure (1000 iterations) was performed to examine the stability of the predictor selection and regression coefficients. In addition, a leave-one-out (jackknife) sensitivity analysis was conducted, in which the regression model was repeatedly estimated after excluding one observation at a time. The consistency of selected predictors and the stability of regression coefficients across these iterations were used to evaluate model robustness.

The type 1 error level was accepted as “*p* < 0.05” for all statistical analyses.

## 3. Results

Table 1 shows the general characteristics of the participants. Of the participants, 54.2% were female, and the mean age was 14.0 ± 1.3 years. The average household income was 3.8 ± 1.9 times the minimum wage in 2024. Regarding the crimes exposed, 29.2% were subjected to injury, 19.4% were subjected to insult or threat, and 51.4% were subjected to sexual offenses. The percentage of participants who had lost at least one of their parents was 13.9%. In addition, 40.3% of the participants were living in a broken family. The percentage of participants with a family history of crime was 31.9%. Lastly, the mean KADS score was determined as 7.3 ± 8.2.

The nutritional parameters of the participants (anthropometric measurements, dietary intakes, duration of breastmilk intake, and KIDMED scores) are given in Table 2. The mean body weight, BMI, BMI-Z score, and body fat percentage were 60.3 ± 9.3 kg, 24.4 ± 3.5 kg/m^2^, 1.36 ± 0.87, and 21.7 ± 7.2%, respectively. Furthermore, the mean dietary energy, carbohydrate, protein, fat, β-carotene, retinol, vitamin D, vitamin E, folate, vitamin B_12_, vitamin C, calcium, iron, and fiber amounts obtained from the three-day dietary records were 2314.1 ± 926.2 kcal, 274.0 ± 160.6 g, 79.1 ± 20.6 g, 97.6 ± 37.2 g, 2940.5 ± 4710.7 µg, 1376.9 ± 5074.0 µg, 4.4 ± 10.6 µg, 17.2 ± 9.7 µg, 316.7 ± 132.5 µg, 6.6 ± 10.8 µg, 109.4 ± 78.2 mg, 745.6 ± 242.3 mg, 12.7 ± 4.9 mg, and 20.6 ± 7.8 g, respectively. In addition, the mean duration of breast milk intake was 13.9 ± 7.5 months and the mean KIDMED score was 3.6 ± 5.1. According to the KIDMED scores, 50.0% of the participants had a low level of Mediterranean diet adherence, while 40.3% had a high level of adherence.

Table 3 shows the results of the correlation analysis between the KADS scores and nutritional parameters. The KADS scores were positively correlated with all anthropometric measurements (*p* < 0.05). In addition, the dietary parameters revealed that the energy, carbohydrate, protein, and fat intake levels were positively correlated with the KADS scores (*p* < 0.05). The dietary vitamin E and iron intake levels were also positively correlated with the KADS scores (*p* < 0.05). In contrast, the dietary vitamin C and fiber intake levels were negatively correlated with the KADS scores (*p* < 0.05). Other parameters found to be negatively correlated with the KADS scores were the duration of breastmilk intake and KIDMED scores (*p* < 0.05).

Stepwise linear regression analysis was performed to determine the nutritional factors associated with the KADS scores (Table 4). The candidate variables examined were body weight, BMI-Z score, body fat percentage, dietary energy, carbohydrate, protein, fat, duration of breastmilk intake, and KIDMED score. According to the *p*-values, the model included KIDMED scores in the first step (model 1), body fat percentage in the second step (model 2), and body weight in the last step (model 3) (variables with *p* > 0.100 were not included in the final model). The KIDMED score (model 1) explained 73.3% of the variation in the KADS score. A lower KIDMED score was associated with a higher KADS score (B = −1.368, *p* < 0.001). The KIDMED score and body fat percentage (model 2) explained 80.5% of the variation in the KADS score. According to model 2, in contrast to the KIDMED score, a high body fat percentage was associated with a high KADS score (B = −1.077, *p* < 0.001 for KIDMED score; B = 0.366, *p* < 0.001 for body fat percentage). Adding body weight to the model, along with the KIDMED score and body fat percentage (model 3), explained 82.2% of the variation in the KADS score. According to model 3, a higher body weight was associated with a higher KADS score (B = −0.883, *p* < 0.001 for KIDMED score; B = 0.375, *p* < 0.001 for body fat percentage; B = 0.155, *p* = 0.012 for body weight). Sensitivity analyses showed that the same variables were retained across bootstrap samples and leave-one-out iterations, with minimal variation in the regression coefficients.

## 4. Discussion

Children are evaluated in judicial processes not only due to their involvement in crime but also due to their exposure to crime. Victimization of crime in children is a consequence of neglect and abuse. It is known that behavioral, psychological, and social abilities are particularly affected in children exposed to domestic violence. This situation may affect feeding behavior, and thus interrupt growth and development [28]. It is highly likely that self-regulatory coping strategies against mood disorders in childhood traumas are associated with adverse health outcomes such as inadequate and unbalanced nutrition [29]. Low self-esteem and the need to cope with negative emotions resulting from criminal victimization may play a role in the development and maintenance of eating disorders, which may occur as an emotional response [30]. Indeed, the negative effects of adverse childhood experiences on overall health are attributed to two main etiologies: chronic stress and compensatory risky behaviors (self-help attempts through substances and foods) [31]. Based on all this relevant information, in the current study, we focused on the relationship between the level of depression and nutritional status in children who experienced traumatic events that constitute crimes such as physical, emotional, and sexual abuse.

The current findings show that body weight, BMI, BMI-Z score, and body fat percentage are highly correlated with depression severity, as assessed by the KADS, in child victims of crime. The association of body weight and body fat percentage with depression was also confirmed by stepwise linear regression analysis. Higher body weight, BMI, BMI-Z score, and body fat percentage measurably indicate overweight and obesity. In the literature, there is no study that evaluated nutritional status in a sample of children and/or adolescents with recorded criminal victimization. On the other hand, the literature provides ample evidence on the relationship between childhood trauma and nutritional status; however, most of these studies were conducted on adults with a history of childhood trauma. One study on children found that acute and chronic malnutrition was more common in girls who had been physically abused, and overweight/obesity was more common in girls who had been sexually abused [32]. Another study reported that physical, sexual, and emotional abuse was very common among Afghan adolescent girls and that these childhood traumas were associated with being malnourished or overweight [33]. Apart from these two reports, the other reports were studies conducted on adults and focused on the relationship between a history of childhood trauma and nutrition-related findings in later life. These studies reported that adverse childhood experiences were associated with obesity, food addiction, emotional eating, uncontrolled eating, and binge eating [29,30,34,35,36,37,38,39,40]. However, the relationship between depressive symptoms and nutritional status was mentioned in a few of these studies. Dedert et al. [40] reported that depressive symptoms and stress disorder were associated with obesity in women exposed to childhood trauma. Michopoulos et al. [29] showed that childhood emotional abuse is associated with emotional eating behavior in adulthood, with depressive symptoms fully mediating this relationship. These results partially support the findings of the current study. We found that obesity-related parameters were associated with depression severity in children and adolescent victims of crime. Notably, the relatively high explained variance observed in this study may reflect the strong interrelationships between dietary quality and anthropometric parameters within this specific high-risk population, rather than model overfitting alone.

The present findings show that depression is associated with intake levels of dietary energy and certain nutrients. The finding that dietary energy, carbohydrate, protein, and fat intake levels are positively associated with depression severity suggests that participants who consume more foods rich in saturated fatty acids (red meat, fast foods, etc.) and simple carbohydrates (fast foods, deserts, sugary drinks, etc.) are more likely to have depressive symptoms. Dietary iron intake levels were positively correlated with depression severity, suggesting a high level of red meat consumption in participants with depressive symptoms. On the other hand, vitamin C and fiber intake levels were negatively associated with depression severity. In other words, it is highly likely that participants with more severe depressive symptoms had insufficient consumption of fruits and vegetables. In fact, studies in adult samples have repeatedly reported that high intakes of foods rich in saturated fats and simple carbohydrates and low intakes of vegetables and fruits are associated with depressive symptoms and other mental health problems [41,42,43,44,45,46,47]. Similarly, our findings suggest the existence of such a relationship in a sample of traumatized children and adolescents victimized by crime. It should also be noted that dietary intake in our study was assessed using a three-day food diary, a method commonly used in pediatric nutrition research. Previous studies have shown that short-term food records can provide reasonably valid estimates of energy and nutrient intake in children and adolescents when appropriate guidance and supervision are provided [48]. Nevertheless, it is important to acknowledge that such methods are subject to reporting bias, including under- or over-reporting, as well as intra-individual variability, particularly in vulnerable populations exposed to psychosocial stress.

In the current study, the low mean KIDMED scores and low compliance of 50.0% of the participants to the Mediterranean diet indicate that the diet quality is very poor in the sample of child and adolescent victims of crime. In a large population study, adverse childhood experiences were found to be significantly associated with a poor diet quality, even after adjustment for ethnicity, gender, and income levels [49]. This finding supports our results. In addition, our findings show that there is a very high negative correlation between the levels of Mediterranean diet adherence and depression severity. This is indeed a remarkable finding and points to the importance of improving eating habits to support mental health in children and adolescents victimized by crime.

Several alternative explanations may account for the observed associations. For instance, emotional eating behaviors, which are commonly reported in individuals exposed to psychological stress or trauma, may contribute to both increased energy intake and higher body weight, thereby indirectly relating to depressive symptoms. In addition, socioeconomic conditions may influence the dietary quality and access to healthy foods, which, in turn, can affect the nutritional status and mental health outcomes. Furthermore, psychological coping mechanisms developed in response to trauma may shape eating behaviors and lifestyle patterns. Therefore, the observed relationships are likely multifactorial and should not be interpreted as direct causal pathways.

Eating disorders and poor diet quality are known risk factors for many chronic diseases [50,51]. Stress-induced eating, which emerges as a coping behavior with negative emotions in individuals with a history of childhood trauma, may be an important pathway for chronic diseases [52]. There are studies emphasizing that childhood traumas are associated with the risk of developing metabolic syndrome; furthermore, levels of malondialdehyde (MDA), a marker of oxidative activity, and C-reactive protein (CRP), a marker of chronic inflammation, are higher in individuals with a history of childhood trauma [52,53,54,55]. Moreover, obesity has been reported to mediate the relationship between childhood trauma and high CRP levels [54]. Therefore, early identification and treatment of disordered eating patterns, together with the promotion of healthy dietary behaviors during childhood and adolescence, are critical for preventing long-term physical and mental health consequences. Our findings underscore the potential value of integrating nutritional education and dietary counseling into psychological interventions for traumatized children as a complementary strategy to improve both metabolic and mental health outcomes. However, further studies are warranted to elucidate the neuropsychobiological mechanisms linking childhood trauma, stress-related eating behaviors, and chronic disease risk.

Several limitations of this study should be acknowledged. First, the cross-sectional design precludes any inference of causality, and the observed associations between nutritional parameters and depression severity should be interpreted with caution. The absence of a comparison (control) group further limits the ability to contextualize the findings and to assess whether the observed associations are specific to this population. Second, the study was conducted in a single forensic medical center with a relatively small sample size (*n* = 72), which may limit the generalizability of the findings to broader populations and different cultural contexts. In addition, the reduction in the sample from 116 eligible participants to 72 included individuals—based on parental consent—may have introduced selection bias. It is possible that families who agreed to participate differ systematically from those who declined, which could influence the observed associations. Third, although only the most relevant nutritional parameters were selected as candidate variables for the regression analysis, the sample size may still be considered modest for multivariable analyses. To address this, a stepwise regression approach was used, resulting in a more parsimonious final model including a limited number of variables. Sensitivity analyses (bootstrap and leave-one-out procedures) indicated that the identified variables were stable despite the relatively small sample size. Importantly, potential confounding variables were not fully controlled in the regression model due to sample size limitations, and therefore, residual confounding may have influenced the observed associations.

## 5. Conclusions

Poor diet quality and adverse body composition were significantly associated with depression severity in child and adolescent victims of crime. These findings highlight the potential relevance of nutritional status in understanding depressive symptoms within this vulnerable population. Importantly, the observed associations do not imply causality and may be influenced by residual confounding factors. Nevertheless, the findings should be interpreted as exploratory, and further longitudinal and interventional studies with larger and more diverse samples are needed to confirm these results and to clarify the directionality of the relationships.

## Figures and Tables

**Table 1:** General characteristics of the participants.

	$\bar{x}$ ± SD	*n* (%)
**Age (years)**	14.0 ± 1.3	
**Gender**		
Female		39 (54.2)
Male		33 (45.8)
**Household income (×minimum wage)**	3.8 ± 1.9	
**Type of criminal act suffered**		
Wounding		21 (29.2)
Insult/threat		14 (19.4)
Crimes against sexual inviolability		37 (51.4)
**Loss of a parent**		
Yes		10 (13.9)
No		62 (86.1)
**Parental cohabitation status**		
Living together		43 (59.7)
Broken family (loss or separation)		29 (40.3)
**Family history of crime**		
Yes		23 (31.9)
No		49 (68.1)
**KADS scores**	7.3 ± 8.2	

$\bar{x}$: mean, SD: standard deviation, KADS: Kutcher Adolescent Depression Scale.

**Table 2:** Nutritional characteristics of the participants.

	$\bar{x}$ ± SD	*n* (%)
**Anthropometric measurements**		
Body weight (kg)		60.3 ± 9.3
BMI (kg/m^2^)		24.4 ± 3.5
BMI-Z score		1.36 ± 0.87
Body fat percentage (%)		21.7 ± 7.2
**Dietary intakes**		
Energy (kcal)		2314.1 ± 926.2
Carbohydrate (g)		274.0 ± 160.6
Protein (g)		79.1 ± 20.6
Fat (g)		97.6 ± 37.2
β-Carotene (µg)		2940.5 ± 4710.7
Retinol (µg)		1376.9 ± 5074.0
Vitamin D (µg)		4.4 ± 10.6
Vitamin E (µg)		17.2 ± 9.7
Folate (µg)		316.7 ± 132.5
Vitamin B_12_ (µg)		6.6 ± 10.8
Vitamin C (mg)		109.4 ± 78.2
Calcium (mg)		745.6 ± 242.3
Iron (mg)		12.7 ± 4.9
Dietary fiber (g)		20.6 ± 7.8
**Duration of breast milk intake (months)**		13.9 ± 7.5
**KIDMED scores**		3.6 ± 5.1
**KIDMED groups** **(Adherence to the Mediterranean diet)**		
Low level	36 (50.0)	
Moderate	7 (9.7)	
High level	29 (40.3)	

$\bar{x}$: mean, SD: standard deviation, BMI: body mass index, KIDMED: Mediterranean Diet Quality Index.

**Table 3:** Correlation of KADS scores with anthropometric measurements, dietary energy and nutrient amounts, duration of breast milk intake, and KIDMED scores.

	KADS Scores	
	r ^1^	*p*-Value
**Anthropometric measurements**		
Body weight	0.655	<0.001
BMI	0.811	<0.001
BMI-Z score	0.686	<0.001
Body fat percentage	0.703	<0.001
**Dietary intakes**		
Energy	0.732	<0.001
Carbohydrate	0.723	<0.001
Protein	0.424	<0.001
Fat	0.523	<0.001
β-Carotene	−0.227	0.055
Retinol	0.126	0.293
Vitamin D	−0.223	0.060
Vitamin E	0.369	0.001
Folate	−0.007	0.951
Vitamin B_12_	0.027	0.820
Vitamin C	−0.359	0.002
Calcium	0.066	0.583
Iron	0.569	<0.001
Dietary fiber	−0.247	0.037
**Duration of breast milk intake (months)**	−0.744	<0.001
**KIDMED scores**	−0.856	<0.001

^1^ Pearson’s rho correlation, KADS: Kutcher Adolescent Depression Scale, BMI: body mass index, KIDMED: Mediterranean Diet Quality Index.

**Table 4:** Multiple linear regression analysis of the variables associated with KADS scores.

KADS Score		B	SEM	β	t	*p*-Value	95% CI	Tolerance	VIF
**Model 1**	Constant	12.649	0.666		18.980	<0.001	11.429–13.870		
	KIDMED scores	−1.418	0.103	−0.867	−13.815	<0.001	−1.615–−1.221	1.000	1.000
**Model 2**	Constant	3.705	2.018		1.758	0.084	−0.003–7.412		
	KIDMED scores	−1.104	0.115	−0.675	−9.604	<0.001	−1.309–−0.899	0.684	1.463
	Body fat percentage	0.352	0.080	0.310	4.418	<0.001	0.207–0.497	0.684	1.463
**Model 3**	Constant	−6.835	4.893		−1.397	0.168	−15.476–1.806		
	KIDMED scores	−0.886	0.144	−0.541	−6.141	<0.001	−1.133–−0.639	0.435	2.299
	Body fat percentage	0.381	0.078	0.336	4.896	<0.001	0.241–0.520	0.682	1.466
	Body weight	0.150	0.063	0.174	2.368	0.021	0.031–0.270	0.562	1.779

Model 1: R^2^ = 0.733, adjusted R^2^ = 0.729 (*p* < 0.001); Model 2: R^2^ = 0.805, adjusted R^2^ = 0.799 (*p* < 0.001); Model 3: R^2^ = 0.822, adjusted R^2^ = 0.814 (*p* < 0.001), Durbin-Watson: 1.223; SEM: standard error of mean, CI: confidence interval for B, VIF: variance inflation factor.

## Data Availability

The data presented in this study are available on request from the corresponding author due to ethical and privacy restrictions related to the sensitive nature of the data involving child and adolescent victims of crime.
